# Study on Mechanical Properties of Permeable Polymer Treated Loess

**DOI:** 10.3390/ma15196647

**Published:** 2022-09-25

**Authors:** Weifan Zhao, Chengchao Guo, Chaojie Wang, Yuke Wang, Lina Wang

**Affiliations:** 1 College of Water Conservancy Engineering, Zhengzhou University, Zhengzhou 450001, China; 2 National Local Joint Engineering Laboratory of Major Infrastructure Testing and Rehabilitation Technology, Zhengzhou 450001, China; 3 Collaborative Innovation Center of Water Conservancy and Transportation Infrastructure Safety, Zhengzhou 450001, China; 4 School of Civil Engineering, Sun Yat-sen University, Guangzhou 510275, China

**Keywords:** grouting, permeable polymer, loess, mechanical properties, durability, microscopic tests

## Abstract

The reinforcement and durability of loess are of great importance for road performance. In this study, a self-designed grouting system and newly permeable polymers were adopted to investigate the mechanical properties and durability of solidified loess (SL), considering different dry densities and water contents. The unconfined compression test and piezocone penetration (CPTU) test were used to examine the mechanical properties. The mechanism of the loess solidified by permeable polymer was analyzed from the micro-level by SEM, MIP, and XRD tests. The test results show that the effect of polymer grouting is obvious, the unconfined compressive strength (UCS) of the SL after grouting is as high as 3.05–5.42 MPa; it is 11.83–20.99 times that of unsolidified loess (UL). The UCS of the SL after grouting is inversely proportional to the dry densities and water contents. After 56 days of immersion, the SL still shows a high compressive strength. The additional erosion of the SL was not caused by the salt solution; the durability is significantly better than that of cement mixing soil. The sensitivity of various factors on the UCS of the SL are service environment > water content > dry density. The SEM tests clearly show that the gel formed by the reaction of the polymer with water on the surface of soil particles makes the bond of soil particles tighter. It can be observed from the MIP test that the cumulative mercury of SL was 0.115 mL/g, which was 33.72% of UL (0.341 mL/g), and the cumulative mercury of SL after immersion in water and salt solutions was 0.183 mL/g and 0.175 mL/g, which was 53.7% and 51.3% of UL (0.341 mL/g), respectively. The XRD results show that there are no other new mineral components produced after grouting and the spacing between crystalline planes decreases, which proves that permeable polymer grouting makes the soil denser and does not erode the soil particles.

## 1. Introduction

Collapsible loess is a kind of special soil widely distributed in Northwest China. The structure of the loess will be destroyed and the bearing capacity will decrease after being soaked in water. Disasters, such as subsidence and landslides, are likely to occur under the action of external forces [1,2]. In recent years, with development in the west and the implementation of the “one belt and one road” strategy, an increasing number of high-speed trains and expressways have been built in the loess area [3]. Consequently, collapsible loess subgrade reinforcement has become an urgent problem for road construction in Northwest China. Loess has typical water-sensitive characteristics and can maintain a relatively stable bearing capacity under waterless conditions. However, once eroded by water, the structure of loess will change and the bearing capacity will decrease rapidly, which will ultimately lead to subgrade collapse or soil slide [4]. Loess is wetted by water under self-weight or additional stress and its structure is destroyed and deformed, which is called “collapsing” [5]. In light of this, the loess must be reinforced.

When the external force is greater than the cohesive force and internal friction force between the soil particles, they will slide in relation to one another eventually leading to shear failure. Therefore, the key function of soft soil reinforcement is to increase the cohesive force between soil particles and improve their ability to resist external forces. Commonly used treatment methods for collapsible loess foundation include the replacement cushion method, CFG pile method, high-pressure jet grouting pile method, and chemical reinforcement method [6]. Chemical reinforcement is a very important reinforcement method. Presently, an increasing number of mechanical property enhancers are used, such as lime, cement, nanoparticles, fly ash, water glass, polypropylene fiber, and calcium lignosulfonate [7,8,9,10,11,12,13]. A large number of scholars have added different modifiers to the soil to explore the consequent property changes of the reinforced soil. Bouhicha et al. [14] solidified the soil by adding a different proportion of barley straw into the soil. The effects of different fiber lengths and fiber components on shrinkage, compressive strength, bending strength, and shear strength were studied. Suat et al. [15] solidified clay by adding waste tires and synthetic fibers to explore the strength and dynamic characteristics of the modified clay. The test results show that waste tire rubber, polyethylene, and polypropylene fibers can be successfully used as reinforcing materials for clay modification. Zhong et al. [16] improved the loess by adding fly ash and analyzed the dynamic residual deformation and dynamic strength evolution characteristics of the improved loess by dynamic triaxial test. Ren et al. [17] studied the reinforcement mechanism and durability of loess reinforced by SH and lime. Li et al. [18] solidified silt with polymer, and studied the effects of polymer content, water content, and soaking time on compressive strength. Chen et al. [19] studied the feasibility of using red mud waste as loess subgrade filling and the mechanical characteristics of red mud-treated loess with a small amount of cement. Lv et al. [20] studied the relationship between the mechanical properties of sulfate saline soil solidified with an alkali-activated geopolymer with the sulfate salt content. Vipulanandan [21] treated the expansive soil with a water-soluble acrylamide polymer solution (PS) and found that the liquid limit (LL) and plasticity index (PI) of the expansive soil decreased by 33% and 57%, respectively. The free swelling of expansive soils was reduced by 60% and the compacted (ASTM 698 standard method) compressive strength increased from 126 kPa to 496 kPa. In addition, the optimum moisture content of soils was reduced. Due to the low shear strength characteristics of sandy soils, Wei [22] investigated the reinforcement properties of a mixture of natural fibers and polymers for sandy soils. The test results showed that the unconfined compressive strength of the specimens increased with increasing fiber and polymer admixture, and in addition, the ductility of the specimens was significantly improved. The water-based polyurethane significantly improved the intergranular cohesion. Jiang [23] prepared five solids in different proportions using sodium hydroxide and water glass as alkali stimulants, mixed with metakaolin and fly ash. The microstructure, mass loss, and compressive strength of the specimens after freeze-thaw cycles were investigated. The test results showed that fly ash helped to suppress the mass loss and radon emission. With the increase of the number of freeze-thaw cycles, the mass loss of the samples gradually increased, the compressive strength gradually decreased and the radon emission rate gradually increased. Yuan [24] studied the microscopic mechanism of action and impact resistance of glass fiber and liquid-modified polyvinyl alcohol polymer (SH polymer) curing granite residual soil. The test results showed that the soil particles were tightly bound under 3.5% content of SH polymer, and the impact resistance of the specimens reached the maximum when the glass fiber content was 3.0%. Hu [25] investigated the reinforcement mechanism of polypropylene (PP) fiber-reinforced consolidated soil and discussed the effect of fiber parameters on the mechanical properties of consolidated soil. It was finally concluded that PP fibers with a length of 12 mm and a fiber content of 0.3% have the best performance and economic cost. Cabalar [26,27] solidified low plasticity clay and sand soils using xanthan gum biopolymer. The hydraulic conductivity of the sandy soils was found to be reduced by more than three to four orders of magnitude, and in addition, the compressive properties, unconfined compression, and triaxial shear properties of the specimens were improved.

All of the above scholars used the mixing method to strengthen the soil, which is more suitable for new roads. However, for the treatment of existing roads, grouting reinforcement is often more effective. In addition, most of the current studies focus on the durability of cured soil under dry and wet cycles [28,29,30,31], while the decay law of mechanical properties of solidified soil under long-term immersion is less studied.

Therefore, in this paper, a new type of permeable polymer was injected into the loess through a self-designed grouting system, and the mechanical properties of the loess before and after grouting were tested using UCS as well as CPTU. Meanwhile, a long-term immersion test scheme was proposed to study the decay of consolidation properties under extreme environments. Finally, microscopic tests were combined to explore the connection between macro and micro. This study can provide some reference basis for the grouting repair of loess roadbeds in northwest China.

## 2. Experimental Processes

### 2.1. Physical Property Indexes of Loess

The loess used in this test was taken from a construction site in Baiyin City, Gansu Province, with a depth of 0.2–2.0 m. The basic physical property indexes of the soil are illustrated in Table 1.

The screening method was used to analyze the particle size gradation of the soil greater than 0.075 mm. The screening results are listed in Table 2.

The pipette method was used for the part with a particle size less than 0.075 mm, and the results of particle size analysis are shown in Table 3.

**Figure 1:**
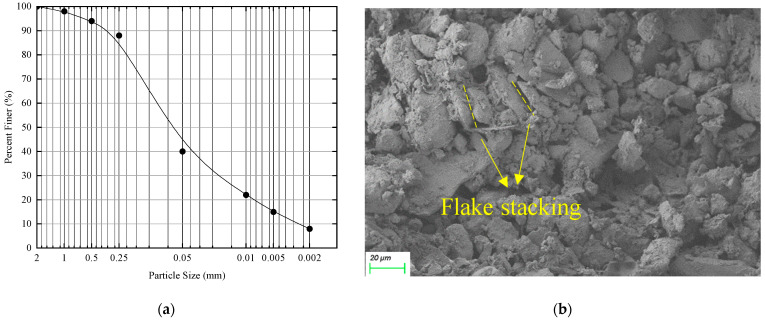
Particle size analysis and microscopic image of tested loess. (**a**) Particle size analysis, (**b**) Microscopic image.

According to the above particle analysis results, the particle size grading curve of soil for this test was drawn, as shown in Figure 1a. The content of particles larger than 0.075 mm in the tested soil is 24% and the plastic index is 5.3. According to the Soil Test Methods for Highway Engineering (JTG E40-2007), it was determined to be low liquid limit powder soil. From Figure 1b, it can be seen that the soil particles are small in size, with mostly face-to-face flake stacking, showing a dispersed structure. This type of soil tends to have collapsibility and should be treated correctly in engineering.

### 2.2. Permeable Polymers

**Figure 2:**
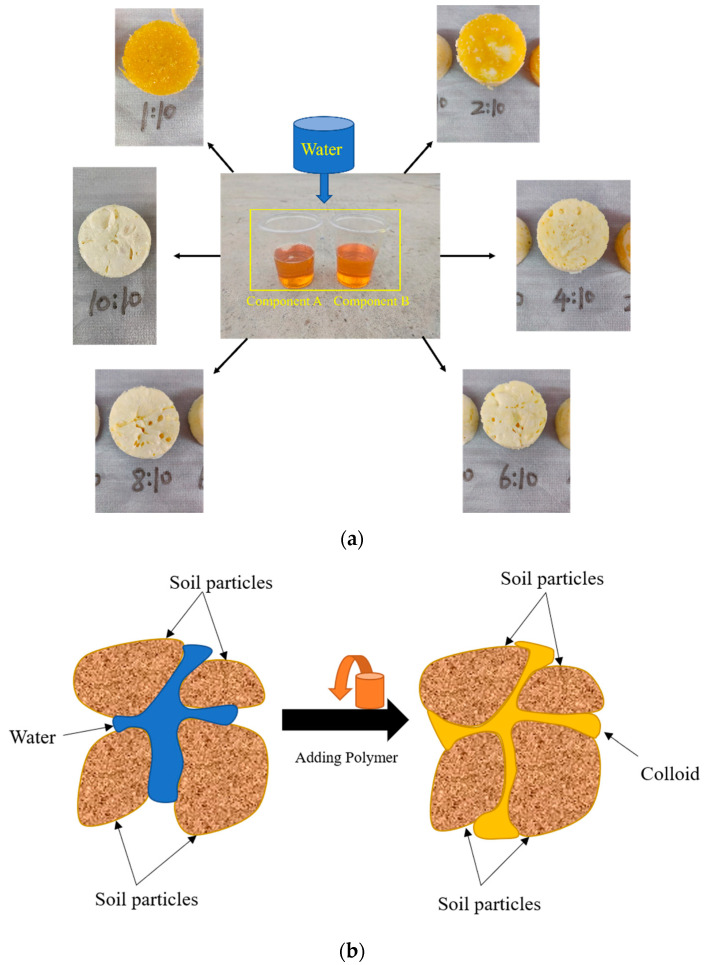
Polymers and solidification mechanism. (**a**) Permeable polymer and hydration products at different water contents. (**b**) Solidification mechanism.

The material for this grouting test is a permeable polymer independently developed by the team, with a slurry density of 1.0–1.2 g/cm^3^ and a gelation time of about 2 h. The permeable polymers are divided into two components, A and B, which are dark yellow and have good fluidity. The chemical composition of permeable polymers is given by Li and Wang [18,32]. Permeable polymer can be fully permeated into the soil and the white colloidal material was produced when the polymer came into contact with water, which could greatly enhance the adhesion between soil particles, thus achieving the purpose of reinforcement. In addition, the structure of the hydration products differed slightly with different water contents. The hydration products of different ratios of polymer with water are shown in Figure 2a. The solidification mechanism is shown in Figure 2b.

The properties of the permeable polymers are illustrated in Table 4.

### 2.3. Specimen Preparation

Combined with the actual dry density and water content of the loess at the borrow site given in Table 1, the dry densities were set as 1.2 g/cm^3^, 1.3 g/cm^3^, and 1.4 g/cm^3^, while the water contents were set to 5%, 10%, and 15%.

The obtained loess was air-dried, crushed, and selected through a 2 mm sieve. The dry density and water content required were prepared for the test according to the standard for geotechnical test methods (GB/T50123-2019); the mass of water required was calculated and it was sprayed evenly on the soil after weighing. The soil was placed into sealed bags, then placed in closed containers for 24 h to make the water soak evenly.

Grouting with a self-designed grouting system, which was mainly divided into the following parts: grouting machine, air compressor, grouting pipe, grouting gun, and soil loading mold. Pressure gauges were equipped on the soil loading mold and grouting machine to display the pressure in the mold and grouting pressure, respectively. To avoid the splitting of soil samples during grouting, the grouting pressure should be controlled at 0.1–0.2 MPa.

**Figure 3:**
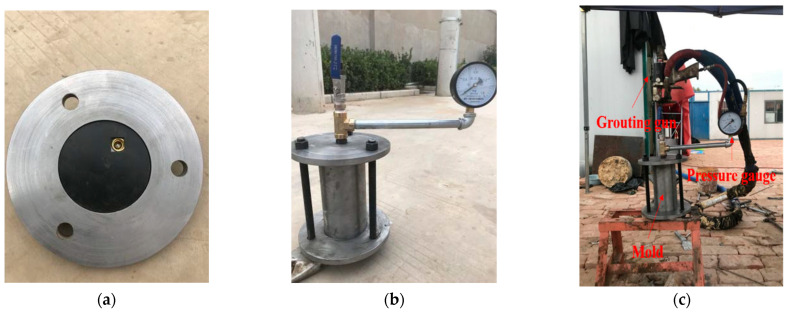

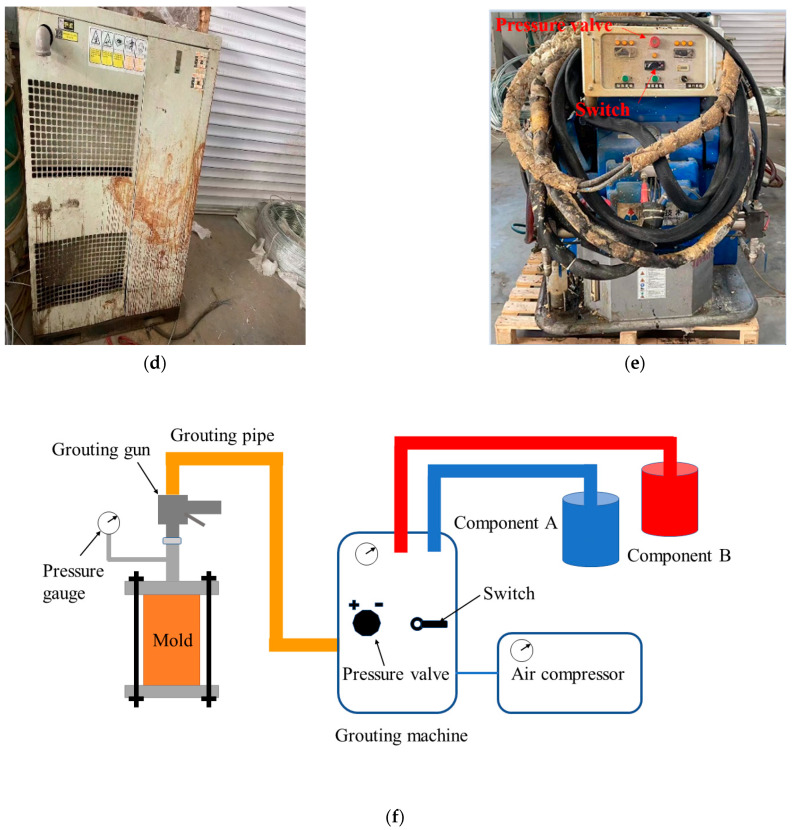
The physical and schematic diagram of the grouting system. (**a**) Sealing rubber gasket, (**b**) Mold, (**c**) Grouting pipe and grouting gun, (**d**) Air compressor, (**e**) Grouting machine, (**f**) Schematic diagram of grouting system.

First, rubber pads were placed in the groove at the bottom and top of the mold for sealing (as shown in Figure 3a). A layer of petroleum jelly was smeared on the inner wall, then, a layer of tin foil was pasted for later demoulding. Bubbles and folds were not allowed during the bonding process. Then, loess was added into the mold five times and compacted evenly. After each layer of soil was compacted, the surface was scraped to avoid obvious stratification. After the soil was filled, the three fixing rods of the mold were tightened through nuts, and then the grouting valve on the top cover of the mold was installed (as shown in Figure 3b). The grouting gun was connected with the top valve, the bottom exhaust valve was opened, and the polymers were slowly injected into the soil. To avoid splitting, the grouting pressure was controlled between 0.1–0.2 MPa and grouting stopped when the pressure reached 0.4–0.5 MPa or when there was slurry leakage, as shown in Figure 3c. After grouting, the top valve was closed, the grouting gun was removed, and it was demoulded after standing for two or three hours. Before demoulding, the mold was flat laid and the top valve was covered with a plastic bag while the residual air pressure and excess slurry in the mold were discharged. Figure 3d,e show the essential air compressor and grouting machine in the grouting system.

Figure 3f shows a schematic diagram of the entire grouting process. After the samples were prepared, they were put into a sealed bag and stored in a constant temperature chamber for standard curing. The dry density, water content, and service environment of the sample were marked on the sealed bag.

### 2.4. Immersion Test

**Figure 4:**
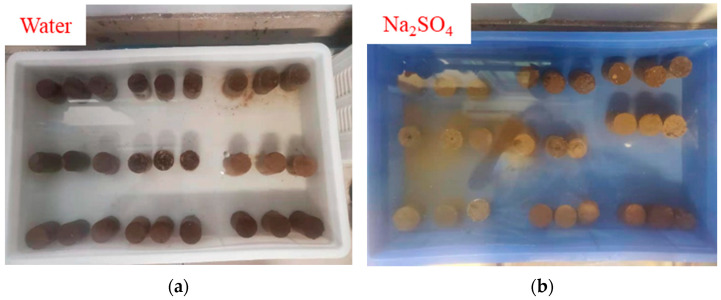
Immersion test. (**a**) Soaked in water, (**b**) Soaked in salt solution.

Partially prepared specimens were immersed in water and salt solution for later determination of their durability. The content of soluble salts varies from region to region. Generally, the content of soluble salts in places with high rainfall is low. Since the average annual rainfall in Gansu is lower, the content of soluble salts in loess there is higher than that in loess from Shaanxi and Shanxi. Therefore, the influence of soluble salt on the SL should be taken into account. The salt selected for this durability test was Na_2_SO_4_ because it is the main soluble salt in Baiyin City, Gansu Province [33], therefore, the SL is most likely to be destroyed by it. Figure 4 shows the sample of the SL immersed in water and salt solution.

### 2.5. Mechanical Tests and Microscopic Tests

The unconfined compression test and piezocone penetration test were used to test the mechanical properties in this study. The unconfined compression test was conducted through the LD-12 Strength Tester of Pavement Materials (STPM) produced by Huanan Instrument Equipment Co., Ltd., Shangyu, China. The sample’s size was 50 mm in diameter × height 100 mm. The pressurization rate was set to 10 mm/min and the *c* and uniformly until the samples were damaged. The calculation is as follows:

(1) $$R_{c}=-\frac{P_{c}}{A}$$

where: *R_c_* is uniaxial compressive strength; *P_c_* is the maximum load on the specimen when it reaches damage; *A* represents the cross-sectional area of the specimen.

The CPTU instrument includes a miniature probe that can provide measurements of cone tip resistance *q_t_* (maximum is 5 MPa), sleeve friction *f_s_* (maximum is 250 kPa), and pore-water pressure *u*_2_ (maximum is 0.5 MPa). DigiDAQ data collector can perform 8-channel high-speed acquisition and the data will be analyzed by the DigiDAQ software (Ver 4.2) which is connected to the laptop computer.

The SEM test instrument was selected from the Sigma300 scanning electron microscope produced by Zeiss, Oberkochen, Germany. The UL and SL of different service environments were scanned in different directions. The arrangement of soil particles and the distribution of polymer gel were observed from the microscopic angle. The D8 advance X-ray diffractometer produced by Bruker company in Germany was used for the XRD test. The phase retrieval and data analysis of XRD test results were carried out through MDI jade software, the changes of material after grouting are qualitatively analyzed, and then the changes of material’s structure after grouting are quantitatively analyzed through the size of crystal plane spacing. The Mercury intrusion porosimetry (MIP) can quantitatively analyze the microscopic pore structure of soil samples and can measure pore sizes in the range of 4 nm–200 μm compared to other measurement methods [34]. When an external force is applied, the mercury overcomes the surface tension and enters the pores of the sample. The size and distribution of the pore of the specimens are calculated according to the principle of balance force, with the expression:

(2) $$P=-\frac{2\sigma \cos\alpha }{r}$$

where: *P* represents the pressure applied to the mercury (N/mm^2^); *σ* is the surface tension coefficient (N/m); *α* represents the impregnation angle of mercury on the sample; *r* is the pore diameter.

In this test, the specimens with dry density *ρ* = 1.3 g/cm^3^ and water content *ω* = 10% were subjected to MIP tests to analyze their pore changes of UL and SL under different service environments.

## 3. Results and Discussion

### 3.1. Comparison of Mechanical Properties between UL and SL

Due to the high strength of the solidified specimen, it is difficult to penetrate the whole of them. Therefore, the specimen was divided into five equal parts, and three different measuring points were selected for each part. The cone tip resistance *q_t_* and sleeve friction *f_s_* of the samples were subject to the average value. The unconfined compression and piezocone penetration test were carried out for the samples and the test results are shown in Table 5.

As shown in Table 5, the mechanical properties of loess were improved by a permeable polymer. The UCS of SL after grouting is 11.83–20.99 times that of UL. From the result of the CPTU test, the cone tip resistance of the SL is 3.09–3.45 times that of the UL, and the sleeve friction is 2.25–3.05 times.

The sample’s mass before grouting was calculated according to the different dry densities and water contents, while the sample’s mass after grouting was weighed by an electronic scale. Three parallel samples were prepared under each condition, and the average value was obtained after weighing the mass, respectively.

It can be seen from Table 6 that the grouting quantity gradually decreases as the dry density of the soil increases. 

Soil is compacted with the increase of dry density, and the arrangement between soil particles is closer, therefore, it is more difficult for the slurry to be penetrated by soil particles, which leads to the decrease of grouting volume. When the dry density is the same, with the increase of water content, the grouting quantity also shows a decreasing trend. Because the soil is a three-phase body composed of soil particles, water, and air, when the pore space between soil particles is certain, the soil voids will be gradually filled with water as the water content increases, and when the voids are all filled with water, the soil is in a water-saturated state. Therefore, when the dry density is certain, the higher the water content is, the more difficult it is for the slurry to be grouted and the smaller the grouting quantity is. These are similar findings to those of Chen and Du’s study [35,36]. Chen found that the curing and penetration effects of modified polyvinyl alcohol (SH) decreased with increasing dry density and were best at dry densities less than 1.5 g/cm^3^. Du found that the amount of slurry injection gradually decreased as the dry density increased.

**Figure 5:**
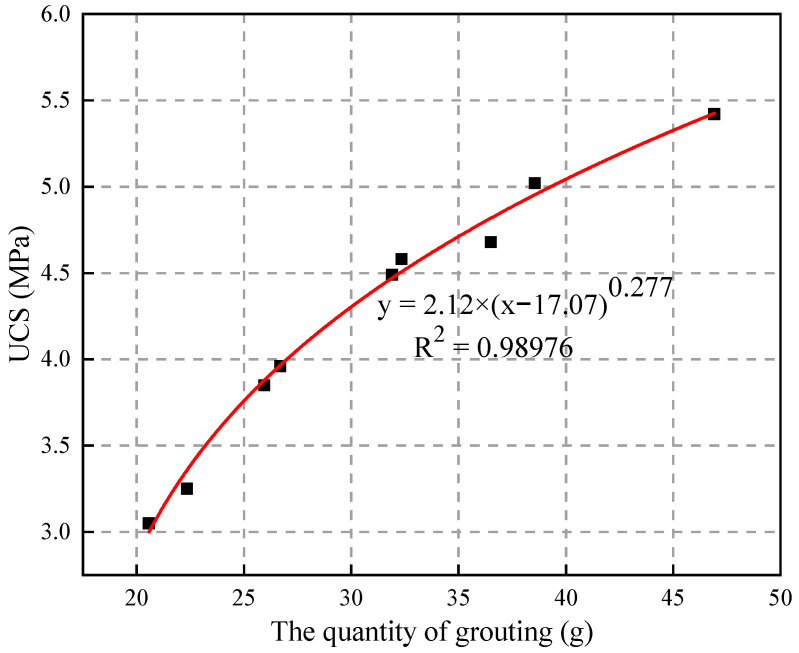
The relationship between grouting quantity and UCS.

From the point of view of grouting quantity and compressive strength, the two have a power–function relationship as a whole; as shown in Figure 5, the larger the grouting quantity, the greater the compressive strength.

### 3.2. Effect of Water Content and Dry Density on the Compressive Strength of SL

**Figure 6:**
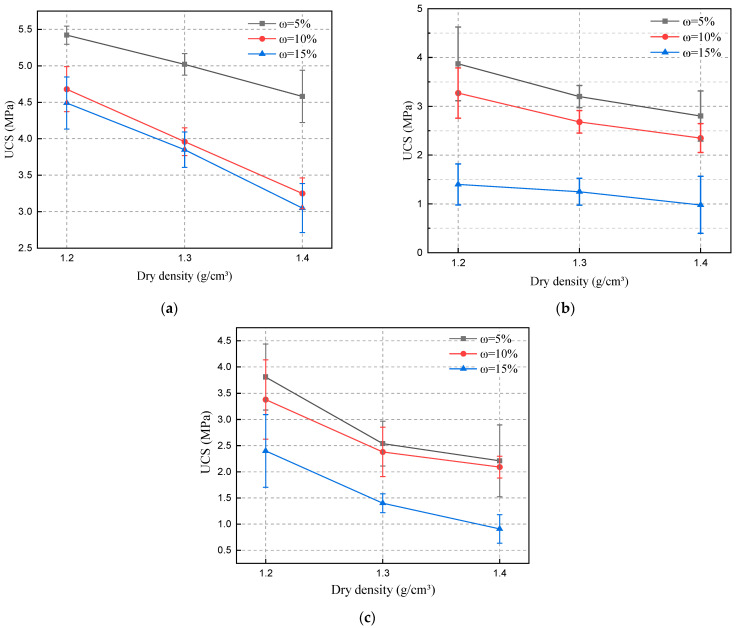
The effect of dry density and water content on compressive strength. (**a**) SL without soaking, (**b**) SL soaked in water, (**c**) SL soaked in salt solution.

The curves of compressive strength shown in Figure 6 were obtained by performing unconfined compressive tests on specimens with different dry densities and water contents.

From Figure 6a–c, it can be seen that the compressive strength of the SL decreases with the increase of the dry density when the water content is certain. The degree of compactness between soil particles is determined by the dry density. The results of Cheng’s study also show that the smaller the compactness of the soil, the greater the grouting volume and the better the reinforcement effect [37]. The larger the dry density, the smaller the spacing between soil particles, and the more difficult it is for slurry to be injected. With the fixed dry density, the compressive strength keeps decreasing with the increase of water content. Pores between soil particles are gradually filled with water due to the increase of water content. As the slurry penetration space is reduced, other slurries become difficult to penetrate and are prone to splitting [38,39].

This problem can be well solved by the permeable polymer used in this test due to its good fluidity. Even when the void space is reduced, the polymer can still penetrate the soil particles and react with the pore water in the soil to produce a gel. The bonding force between the soil particles can be increased by the gel. Due to the increase of water content, the viscoelasticity of the gel generated by the reaction between the polymer and water increases. The shear damage time of SL is prolonged. Compared with slurries such as cement and sodium silicate, the permeable polymer has better permeability and reinforcement ability. Permeable polymer still has a good reinforcement effect despite the increase of dry density and water content.

### 3.3. Effect of the Service Environments on Compressive Strength of SL

Loess is often attacked by rainwater and groundwater in actual projects. The solidified soil can be damaged due to the soluble salts in loess dissolving when the water content increases. To investigate the effect of water and soluble salts on the compressive strength of the solidified soil, this test simulated different service environments, and the solidified soil was soaked in water and salt solution, respectively. The immersion time was set up to 56 days to accelerate the damage of SL and to investigate its durability under extreme conditions.

**Figure 7:**
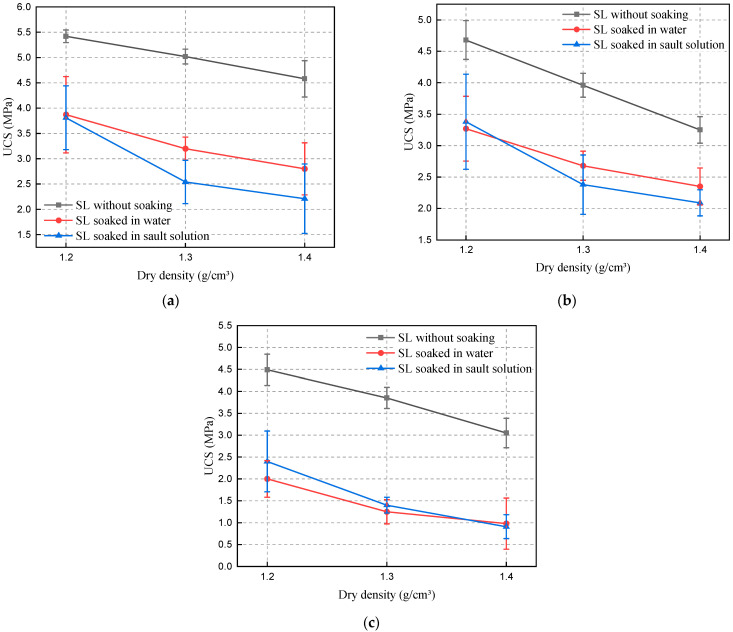
The effect of the service environments on compressive strength. (**a**) 5% water content, (**b**) 10% water content, (**c**) 15% water content.

It can be seen from Figure 7a–c that the compressive strength of the specimens under water and salt solution immersion are close to each other, which proves that the two solutions have the same degree of erosion on the SL, indicating that the polymer gel is not subject to salt erosion. In addition, the standard deviation of the specimens increased after soaking and the variability of the data increased, as can be seen in Figure 7. This may be due to the fact that soaking caused irregular voids as well as cracks inside the specimens, while this erosion phenomenon was random, so that each specimen suffered a slightly different degree of damage. This is similar to the increase in the standard deviation of the residual compressive strength due to cracks inside the high-strength concrete (HSC) after high temperature (20–400 °C) [40].

After 56 days of immersion, the compressive strength of the solidified soil decreased by 0.9–2.6 MPa, still retaining a compressive strength of 0.91–3.87 MPa. The gel generated by the reaction of the polymer with water is not subject to the corrosion of Na_2_SO_4_. In this respect, the corrosion resistance of permeable polymer is better than that of cement slurry [41]. By comparing the reinforcement effect with other chemical pastes, it was found that the reinforcement effect of the SL after soaking is still twice that of the loess with 9% cement content [42], 4.33 times that of the loess treated by F_1_ ion curing agent [43], 5 times that of the loess treated by CaCl_2_, 3.83–15.7 times of the UL.

Through an immersion test and a series of data comparisons, it was found that the compressive strength of the SL after immersion was stronger than the reinforcement effect of the above materials without immersion, and the disadvantages of traditional cement slurry, fly ash, and quicklime, which is susceptible to salt corrosion, were solved [44], therefore, the permeable polymer has better durability and reinforcement effect in loess areas.

### 3.4. Sensitivity Analysis

In this test, three factors were set: dry density, water content, and service environment. Concurrently, three horizontal variables were set under each factor. The method of orthogonal test analysis was used to investigate the factor that had the greatest influence on the compressive strength, and the orthogonal table L9 (3^3^) was set as shown in Table 7.

From Table 7, it can be seen that different service environments, water contents, and dry densities can affect the effect of grouting reinforcement. Hence, the main factors affecting grouting reinforcement were analyzed using the range analysis method; the results are shown in Table 8.

**Figure 8:**
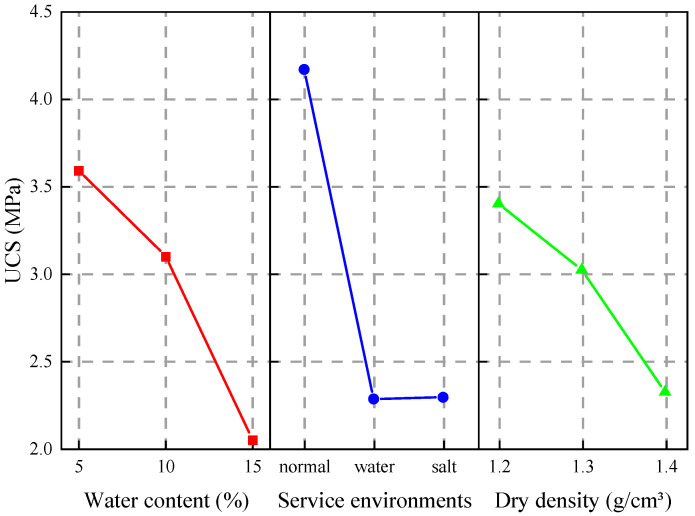
Influence trend of various factors on compressive strength.

The influence trend of various factors on compressive strength is shown in Figure 8. The analysis of the effect of grouting under different working conditions shows that the factors influencing the compressive strength of the solidified soil in order are: service environment > water content > dry density. Yang [45] studied the effects of dry density of loess, water content, water–cement ratio, and grouting pressure on the reinforcement effect, and concluded that water–cement ratio and water content were the main controlling factors. The service environment was introduced in our study. The internal structure of the soil will be damaged and the cohesion between soil particles will be reduced after 56 days of long-time immersion, thus, the compressive strength of the solidified soil after immersion decreases. Since the polymer reacts with water, the water content has a greater effect on compressive strength than dry density. Therefore, the service environment and water content are the main controlling factors.

### 3.5. Analysis of the Damage Forms of SL

**Figure 9:**
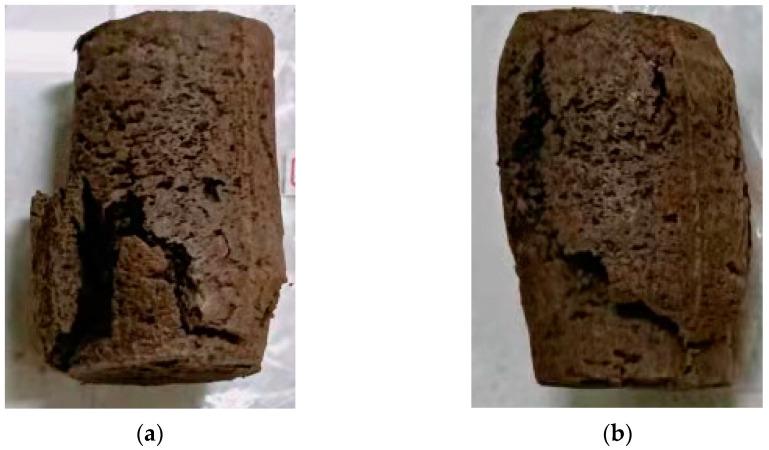
Damage form ⅰ and ⅱ. (**a**) Damage ⅰ, (**b**) Damage ⅱ.

**Figure 10:**
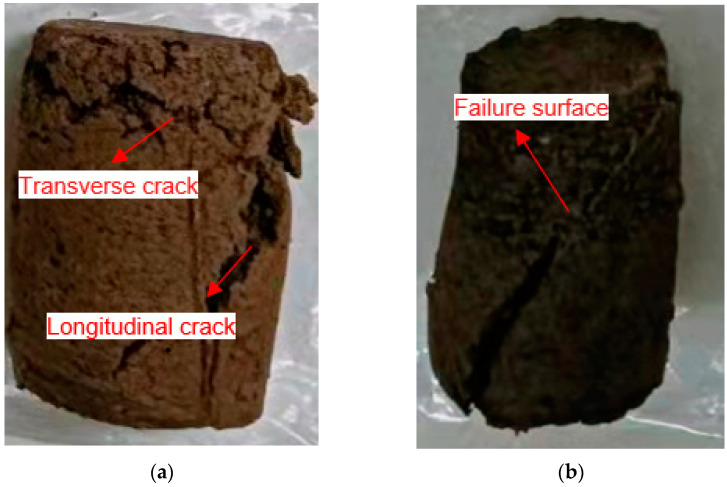
Damage form ⅲ and ⅳ. (**a**) Damage form ⅲ, (**b**) Damage form ⅳ.

A comparative analysis of the damage patterns of the specimens demonstrated that the specimens mainly showed four damage forms, as shown in Figure 9 and Figure 10. The damage forms of SL in this study are similar to that of saline soil mixed with cement and wheat straw, but the difference is that the addition of wheat straw changes the damage behavior of the solidified soil [42].

Damage form ⅰ: as shown in Figure 9a, the fracture starts from the bottom and gradually diffuses upward. When the diffusion height reaches the middle of the sample, it is completely destroyed. This failure generally occurs when the water content is 10%. When the water content is low, the surface of soil particles is mainly covered by the water in the soil to form a water film. When the pores between soil particles are large, the water film does not directly contact each other to form a connection. The interior of the soil will be smoothly penetrated by the slurry. When the water content increases, the water film is connected and even free water is formed, the channel of slurry penetration is blocked by the water, meanwhile, the water in the soil will be transferred to the lower part under the grouting pressure. The result is that the lower part has less strength than the upper part. Therefore, the damage first appeared in the lower part, and then a larger rupture surface was formed from the bottom up, and finally, the whole soil sample was destroyed.

Damage form ⅱ: as shown in Figure 9b, the fracture started from the bottom and diffused upward, and obvious dilatancy occurred in the diffusion process. At this time, the fracture surface continued to spread along the fragile surface inside the soil sample until it penetrated the sample. Generally, when the water content is 15%, the sample will have a dilatancy phenomenon. The lower the dry density, the more obvious the dilatancy phenomenon. Compared with the first failure form, this process takes a long time, has a wide failure area, and a certain ductility is presented in the compression process. The phenomenon of dilatancy is due to the high water content of the specimen, and the ductility and viscoelasticity of the gel were enhanced. Therefore, tensile and tear phenomena will occur during the destruction process, thereby prolonging the failure time.

Damage form ⅲ: as shown in Figure 10a, the structural strength of the specimen was damaged under the transverse and longitudinal cracks. This form of damage with transverse and longitudinal cracks is usually seen in specimens that are soaked. Due to the long-time immersion, the interior of the soil was penetrated by the water from the surface. A few soil particles or agglomerates were gradually dissolved under the water erosion, thus, weak surfaces were formed inside. The irregular transverse and longitudinal cracks will be shown in the later compressive test when the weak surfaces are connected. 

**Figure 11:**
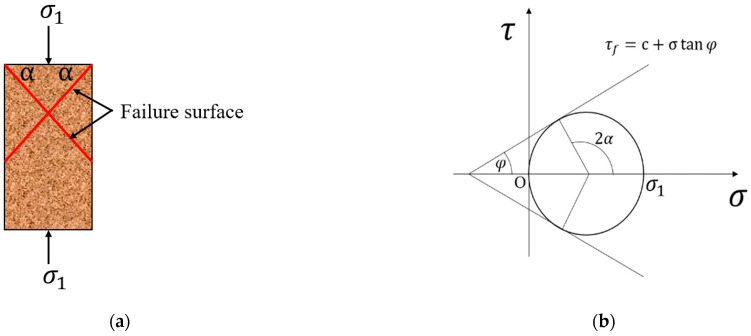
The location of the shear failure surface in the soil. (**a**) Location of the failure surface, (**b**) The destruction Mohr circle and strength wrapping wire.

Damage form ⅳ: as shown in Figure 10b, the specimen is damaged with a certain regular oblique section, which generally occurs when the dry density *ρ* = 1.2 g/cm^3^ and water content *ω* = 5%. In this case, the slurry penetration resistance is small, the strength distribution of the solidified soil is more uniform, and the soil sample will be destroyed along a regular cross-section under the action of axial stress. When the shear stress on a face inside the soil reaches the shear strength, i.e., *τ* = *τ_f_*, the soil is in the ultimate equilibrium stress state. Since the magnitudes of shear and positive stresses on different faces inside the soil are different, the soil reaches the ultimate equilibrium state as long as one face in the soil unit is damaged. The molar circle of the soil sample when it is destroyed under the action of external force *σ*_1_ is drawn in the *τ*-*σ* coordinate axis. According to the Mohr–Coulomb strength theory, the destruction Mohr circle must be tangent to the strength envelope, as shown in Figure 11b. The tangent point corresponds to the damage point, and the damage point corresponds to the location of the damaged surface inside the soil, where the angle is 2*α* = 90° + *φ*/2 between the principal stress *σ*_1_ and the tangent point, so when the positive stress reaches the ultimate stress that the soil can withstand, the specimen is damaged along the cross-section of the active surface of the principal stress *σ*_1_ rotated counterclockwise or clockwise by an angle of *α*, as shown in Figure 11a.

### 3.6. Microstructure Properties of NL and SL 

#### 3.6.1. SEM Analysis

The arrangement of the soil particles and the specific distribution of the gel before and after grouting can be directly observed by the SEM test. The microstructure of the soil can directly affect the macroscopic physical properties, such as strength, permeability, deformation characteristics, etc.

**Figure 12:**
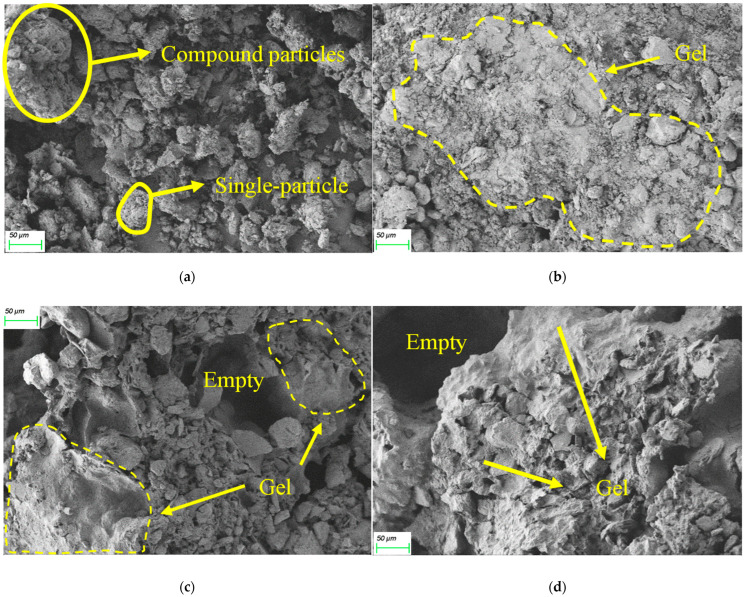
The SEM of UL and SL in different service environments. (**a**) UL, (**b**) SL without soaking, (**c**) SL soaked in water, (**d**) SL soaked in salt solution.

Figure 12a shows the loess before grouting; it can be seen that the soil particles are randomly stacked among each other. There are two forms of soil particles, single-particle, and compound particles. The fine single-particle accounts for the main part. The pores are porous and scattered arrangements and the skeleton of the whole soil is in the form of an overhead pore structure. The soil particles are closely bonded after grouting and the polymer gel in the form of sheets can be observed in Figure 12b to bond the soil particles together. The pores between the soil particles are filled with polymers and the porosity is effectively reduced. The adhesion between soil particles was increased and the porous structure of loess was changed by the permeable polymer. This is consistent with Wang’s observation [32].

Figure 12c,d shows the degree of erosion of water and salt solution for solidified soil, respectively. The bond between the polymer and soil particles was reduced after the long soaking time of 56 days. A few soil particles or soil particle agglomerates were disintegrated after being soaked while the overall soil samples still maintain good integrity. Although the compressive strength of the soaked specimens decreased compared with that of not soaked, the soaked specimens still had strong compressive strength and could meet the road performance. The gel was able to maintain strong cementing ability and viscoelasticity even after soaking, therefore, most of the soil particles still existed in the form of agglomerates.

#### 3.6.2. MIP Analysis 

**Figure 13:**
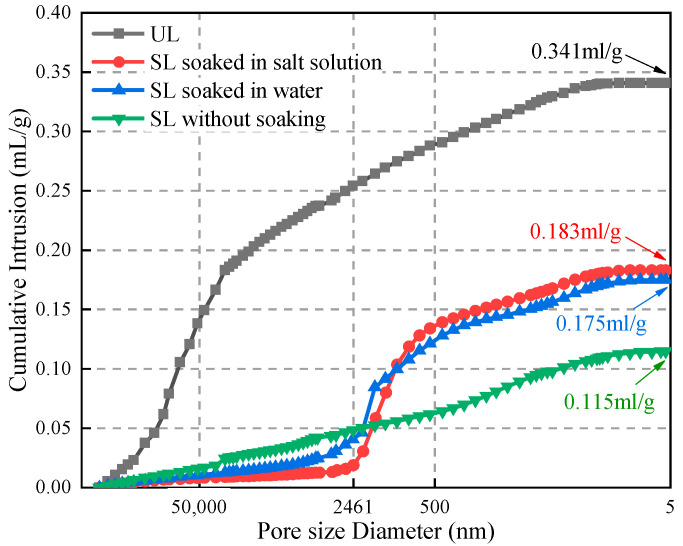
Cumulative intrusion.

The cumulative intrusion amount of mercury can directly reflect the total volume of pores in the sample [46]. The greater the amount of mercury pressed into the sample, the larger the pore volume is. As can be seen in Figure 13, the cumulative intrusion of mercury after grouting is significantly smaller than that before grouting, which proves that the polymer has good fluidity in the soil particles and can penetrate into them to fill the pores.

As the reaction between the polymer and water proceeded, the soil was gradually compacted by the continuously generated gel, which further reduced the content of pores in the soil. The cumulative intrusion of the UL in Figure 13 was higher than that of the SL at each pore size diameter. When the pore size diameter was 5 nm, the cumulative intrusion amount of the UL was 1.94 times that of the SL after soaking, and 2.92 times that of the SL without soaking. From the microscopic point of view, the lower the amount of cumulative intrusion of mercury, the smaller the porosity and the denser the soil. Macroscopically, the greater the compressive strength, the better the mechanical properties. This is in accordance with the results of the unconfined compressive and CPTU test. When the SL was not soaked, there was a linear relationship between the cumulative intrusion and the pore diameters. The absence of abrupt changes in the curve of SL without soaking indicated that the pore structure of the SL was more uniform and there were no major fluctuations in the number of pores at each level of pore diameters. In contrast, the cumulative intrusion of the SL after immersion showed a surge when the pore size reached about 2461 nm and slowed down at 500 nm. This indicated that the long-term immersion in water and salt solutions led to a re-increase in the number of pores in the interval 500–2461 nm. Soil particles in this size range were stripped by solution erosion, leading to the appearance of cavities. Macroscopically, the compressive strength of the specimens and the CPTU test results showed a decrease.

**Figure 14:**
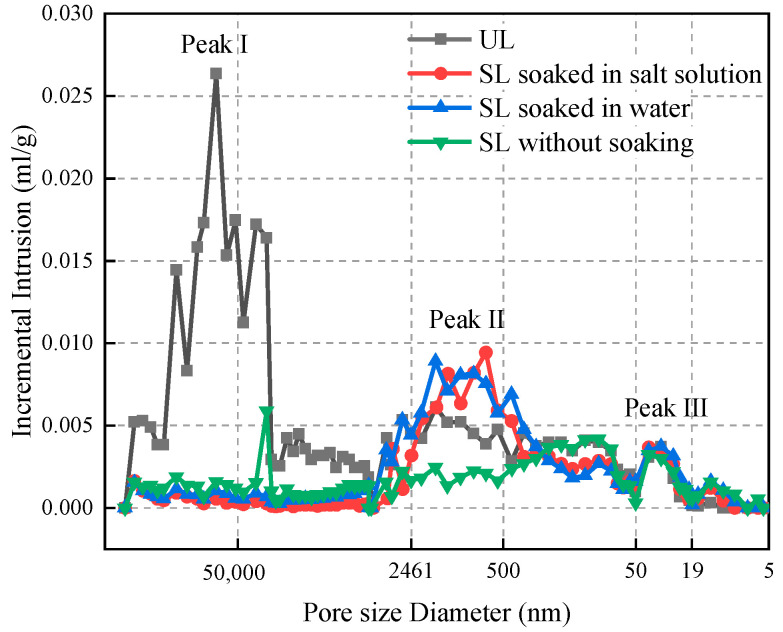
Incremental intrusion.

In Figure 14 we plotted the pore size distribution curve of loess under different conditions.

Among them, the UL showed three marked peaks: macroporosity peak (peak Ⅰ) and microporosity peaks (peak Ⅱ and Ⅲ) [47], while the SL, regardless of whether it was soaked or not, both showed two marked peaks (peak Ⅱ and Ⅲ). For the UL, the macroporosity peak occupies the major part of the pore volume. Because the pores of the UL are mostly macropores and this structure of macropores often leads to its collapsibility. While the pore distribution of the SL is mainly concentrated in microporosity peaks II and III. The permeable polymer effectively filled the macropores in the UL, improving the loose and porous structural characteristics of the loess. The location of Peak II varies depending on the service environment. After soaking the SL, the position of peak II was shifted to a larger pore size. From the above analysis, it can be learned that the main reason for this offset phenomenon is that the immersion of water and salt solution makes some of the soil particles in the 500–2461 nm interval lose their bond with the soil itself, which also directly leads to the decrease of compressive strength of the SL. Peak Ⅲ is common to the unsolidified and SL; the pore diameter in this range is only about 19–50 nm because the pore diameter is too small for the permeable polymer to penetrate into it, therefore, there is no change in the pore volume under this pore diameter.

#### 3.6.3. XRD Analysis

**Figure 15:**
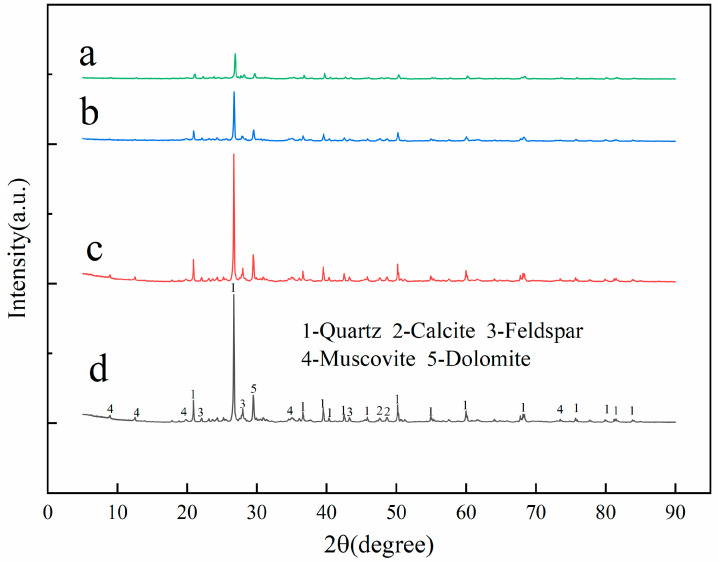
XRD charts: (a) UL; (b) SL without soaking; (c) SL soaked in water; (d) SL soaked in salt solution.

XRD tests were conducted on the UL and SL under three service environments to determine whether the mineral composition of the loess changed and whether the permeable polymer reacted with the soil particles. The MDI jade software was used for qualitative analysis of the test results. It can be seen from Figure 15 that the diffraction peaks of UL and SL under different service environments are the same, indicating that the main mineral composition did not change after grouting. The main components are still quartz, calcite, feldspar, muscovite, and dolomite. The height of the diffraction peaks is different because the diffraction intensity of the material can be affected by the immersion test. The diffraction intensity and the peak value of the soaked samples decreased. It can be explained that there is no new crystal material produced after grouting. The loess was reinforced by the gel produced through the reaction between permeable polymer and water. The cohesion of soil particles was increased and the ability of SL to resist shear failure was enhanced. This is consistent with the results observed by the SEM test. This conclusion is also consistent with Zhu’s findings [48].

The crystal plane spacing of minerals with incident angles of 20°, 40°, and 60° were extracted in MDI jade software [42,43]. The results are shown in Table 9. It can be seen from Table 9 that in both low and high angle areas, when the incident angles are the same, the crystal plane spacing of UL is greater than that of the SL after grouting. The soil particles were arranged more closely and the contact area between them was increased after grouting. The friction and compactness were improved and the compressive strength of the SL was increased.

## 4. Conclusions

In this study, based on the self-developed permeable polymer and grouting system, the loess was solidified under different working conditions, and the mechanical properties and durability of SL were investigated. Combined with SEM, MIP, and XRD tests, the curing mechanism was analyzed. The main conclusions are as follows:

1. The compressive strength of SL is as high as 3.05–5.42 MPa, while the cone tip resistance and sleeve friction increased 3.09–3.45 times and 2.25–3.05 times, respectively. The compressive strength is positively proportional to the grouting volume and inversely proportional to the water content and dry density.
2. Based on the sensitivity analysis, it is concluded that the service environment and water content are the main control factors affecting the reinforcement effect.
3. The damage forms of SL can be summarized as four types:
  i. When the water content is 10%, the cracks are produced from the bottom up;
  ii. When the water content is 15%, the shear expansion occurs in the specimen;
  iii. After immersion, both transverse and longitudinal cracks appear in the soaked specimen;
  iv. When the water content is 5%, the rupture surface is a regular oblique section.
4. Microscopically, no new mineral components were produced in the SL, and the soil particles were mainly cemented by the gel. The cumulative intrusion amount of the UL was 1.94 times that of the SL after soaking, and 2.92 times that of the SL without soaking, indicating a significant decrease in porosity. The porosity of SL increased after soaking, but it was still smaller than UL.

## Figures and Tables

**Table 1:** Physical property indexes of loess.

Type of Soil	Liquid Limit	Plastic Limit	Void Ratio	The Plastic Index	Water Content/%	Dry Density/ g/cm^3^
Loess	24.2	18.9	0.62	5.3	5–14.6	1.42

**Table 2:** The results of particle size analysis of coarse-grained soil.

**Particle Size/mm**	2	1	0.5	0.25	0.075
**Percent Passing/%**	100	98	94	88	76

**Table 3:** The results of particle size analysis of fine-grained soil.

Particle Size/mm	Mass/g	Percent Passing/%
0.05	0.202	40
0.01	0.108	22
0.005	0.074	15
0.002	0.040	8

**Table 4:** Properties of permeable polymers.

Index	Properties
The initial viscosity/mPa·s	≤50
Density/g/cm^3^	1.0–1.2
Compressive strength/MPa	≥20
Gelation time/min	≤120

**Table 5:** Comparison of Mechanical properties.

The Type of Samples	UCS/MPa			*q_t_*/kPa		*f_s_*/kPa	
	UL	SL	Improvement (%)	UL	SL	UL	SL
*ρ* = 1.2 g/cm^3^, *ω* = 5%	0.2465	5.42	2099	93.69	415.16	28.325	114.62
*ρ* = 1.3 g/cm^3^, *ω* = 5%	0.2551	5.02	1868	96.25	396	28.357	102.35
*ρ* = 1.4 g/cm^3^, *ω* = 5%	0.2633	4.58	1639	93.201	391.6	28.389	103.6
*ρ* = 1.2 g/cm^3^, *ω* = 10%	0.2377	4.68	1869	99.25	406.20	28.324	100.258
*ρ* = 1.3 g/cm^3^, *ω* = 10%	0.2431	3.96	1529	92.286	407.65	27.265	98.203
*ρ* = 1.4 g/cm^3^, *ω* = 10%	0.2465	3.25	1218	92.318	411.67	28.235	96.365
*ρ* = 1.2 g/cm^3^, *ω* = 15%	0.2178	4.49	1962	93.52	415.16	26.325	95.23
*ρ* = 1.3 g/cm^3^, *ω* = 15%	0.2281	3.85	1588	95.802	409.11	28.023	91.03
*ρ* = 1.4 g/cm^3^, *ω* = 15%	0.2377	3.05	1183	92.28	410.34	27.365	90.64

**Table 6:** Grouting quantity.

The Type of Samples	The Mass before Grouting/g	The Average Mass after Grouting/g	Average Grouting Quantity/g	UCS/MPa
*ρ* = 1.2 g/cm^3^, *ω* = 5%	247.39	294.31	46.92	5.42
*ρ* = 1.3 g/cm^3^, *ω* = 5%	268.02	306.58	38.56	5.02
*ρ* = 1.4 g/cm^3^, *ω* = 5%	288.63	320.98	32.35	4.58
*ρ* = 1.2 g/cm^3^, *ω* = 10%	259.18	295.69	36.51	4.68
*ρ* = 1.3 g/cm^3^, *ω* = 10%	280.78	306.74	25.96	3.96
*ρ* = 1.4 g/cm^3^, *ω* = 10%	302.38	324.74	22.36	3.25
*ρ* = 1.2 g/cm^3^, *ω* = 15%	270.96	302.87	31.91	4.49
*ρ* = 1.3 g/cm^3^, *ω* = 15%	293.54	320.24	26.7	3.85
*ρ* = 1.4 g/cm^3^, *ω* = 15%	316.12	336.70	20.58	3.05

**Table 7:** Orthogonal test design.

Sample Number	Water Content %	Dry Density g/cm^3^	Service Environment	Compressive Strength/MPa
1	5	1.2	Not soaked	5.42
2	5	1.3	Soaked in salt solution	2.54
3	5	1.4	Soaked in water	2.8
4	10	1.2	Soaked in salt solution	3.38
5	10	1.3	Soaked in water	2.68
6	10	1.4	Not soaked	3.25
7	15	1.2	Soaked in water	1.4
8	15	1.3	Not soaked	3.85
9	15	1.4	Soaked in salt solution	0.91

**Table 8:** Analysis of range table.

Analysis Index (K_i_)	Factor 1 Water Content	Factor 2 Dry Density	Factor 3 Service Environment
K_1_	3.59	3.4	4.17
K_2_	3.10	3.02	2.28
K_3_	2.05	2.32	2.29
D_K_	1.54	1.08	1.89
Sensitivity	Service environment > Water content > Dry density		

Note: K_i_ is the mean of the test results corresponding to level factor i.

**Table 9:** The mineral crystal plane spacing of UL and SL.

Angle of Incidence	Index of Crystal Plane	Samples in Different Conditions			
		UL	SL Without Soaking	SL Soaked in Water	SL Soaked in Salt Solution
20°	(1 0 0)	4.4358	4.4149	4.4293	4.4293
40°	(1 0 2)	2.2522	2.2484	2.2519	2.2518
60°	(1 2 1)	1.5405	1.5395	1.5402	1.5403

## References

[1] Pan L., Zhu J.G., Zhang Y.F. (2021). Evaluation of Structural Strength and Parameters of Collapsible Loess. Int. J. Geomech..

[2] Shao Y., Yuan M., Hao Y., Sun X. (2020). Laboratory Test Research on Collapsible Loess Foundation Treatment. E3S Web Conf..

[3] Shao S., Shao S., Li J., Zhu D. (2021). Collapsible deformation evaluation of loess under tunnels tested by in situ sand well immersion experiments. Eng. Geol..

[4] Hou K., Qian H., Zhang Y., Qu W., Ren W., Wang H. (2021). Relationship between fractal characteristics of grain-size and physical properties: Insights from a typical loess profile of the loess plateau. CATENA.

[5] Zhen Y., Liu B. (2021). Experimental Study on the Performance of Improved Collapsible Loess Mixture with Concrete Crushed Gravel. Earth Environ. Sci..

[6] Zhao Y., Han K., Jiang Y., Mi W., Wu X. (2021). Research on Present Situation and the New Technology About Reinforcement in Collapsible Loess Foundation Inside Tunnels. Eng. Appl. Sci..

[7] An N., Yan C.G., Wang Y.C. (2021). Experimental study on anti-erosion performance of polypropylene fiber-reinforced loess. Rock Soil Mech..

[8] Prakash K.G., Krishnamoorthy A. (2022). Stability of Embankment Constructed on Soft Soil Treated with Soil–Cement Columns. Transportation Infrastructure Geotechnology. Transp. Infrastruct. Geotechnol..

[9] Cong M., Chen L., Chen B. (2014). Analysis of strength development in soft clay stabilized with cement-based stabilizer. Constr. Build. Mater..

[10] Coo J.L., So Z.P.S., Ng C.W.W. (2016). Effect of nanoparticles on the shrinkage properties of clay. Eng. Geol..

[11] Gao Y., Qian H., Li X., Chen J., Jia H. (2018). Effects of lime treatment on the hydraulic conductivity and microstructure of loess. Environ. Earth Sci..

[12] Ran K., Zhang F., Wang G., Peng J. (2018). Stabilization of Loess Using Nano-SiO_2_. Materials.

[13] Li G.Y., Hou X., Mu Y.H., Ma W., Mao Y.C. (2019). Engineering properties of loess stabilized by a type of eco-material, calcium lignosulfonate. Arab. J. Geosci..

[14] Bouhicha M., Aouissi F., Kenai S. (2005). Performance of composite soil reinforced with barley straw. Cem. Concr. Compos..

[15] Akbulut S., Arasan S., Kalkan E. (2007). Modification of clayey soils using scrap tire rubber and synthetic fibers. Appl. Clay Sci..

[16] Zhong X., Wang Q., Liu Z., Bai L., Ma J., Liu F., Li N., Wang J. (2020). Dynamic strength of fly ash-modified loess subgrade under influences of drying-wetting cycle. Chin. J. Geotech. Eng..

[17] Ren X. (2017). Experimental Study on Durability of SH and Lime Reinforced Loess. Ph.D. Thesis.

[18] Li F., Wang C., Xia Y., Hao Y., Zhao P., Shi M. (2020). Strength and Solidification Mechanism of Silt Solidified by Polyurethane. Adv. Civ. Eng..

[19] Chen R., Guo C., Dong X., Mi D., Anand J.P., Duan W. (2019). Mechanical properties and micro-mechanism of loess roadbed filling using by-product red mud as a partial alternative. Constr. Build. Mater..

[20] Lv Q., Jiang L., Ma B., Zhao B., Huo Z. (2018). A study on the effect of the salt content on the solidification of sulfate saline soil solidified with an alkali-activated geopolymer. Constr. Build. Mater..

[21] Vipulanandan C., Mohammed A. (2020). Characterizing the Index Properties, Free Swelling, Stress-Strain Relationship, Strength and Compacted Properties of Polymer Treated Expansive CH Clay Soil Using Vipulanandan Models. Geotech. Geol. Eng..

[22] Wei J., KFang Liu J., Chen Z., Kanungo D.P., Lan X., Jiang C., Shi X. (2018). Effect of Sisal Fiber and Polyurethane Admixture on the Strength and Mechanical Behavior of Sand. Polymers.

[23] Jiang F., Hao Y., Wu H., Liu Y., Wang Z., Tan B., Zhang C., Lan M. (2022). Study on damage degradation and radon emission from uranium tailing polymer-solidified soil under freeze-thaw cycles. J. Radioanal. Nucl. Chem..

[24] Yuan B., Li Z., Chen Y., Ni H., Zhao Z., Chen W., Zhao J. (2022). Mechanical and microstructural properties of recycling granite residual soil reinforced with glass fiber and liquid-modified polyvinyl alcohol polymer. Chemosphere.

[25] Hu C., Weng X., Liu C., Jiang L., Liu J., Li W. (2021). Performance of Polypropylene Fiber-Reinforced Solidified Soil. Adv. Civ. Eng..

[26] Cabalar A.F., Awraheem M.H., Khalaf M.M. (2018). Geotechnical Properties of a Low-Plasticity Clay with Biopolymer. J. Mater. Civ. Eng..

[27] Cabalar A.F., Wiszniewski M., Skutnik Z. (2017). Effects of Xanthan Gum Biopolymer on the Permeability, Odometer, Unconfined Compressive and Triaxial Shear Behavior of a Sand. Soil Mech. Found. Eng..

[28] Mahedi M., Cetin B., Cetin K.S. (2019). Freeze-thaw performance of phase change material (PCM) incorporated pavement subgrade soil. Constr. Build. Mater..

[29] Zhang Q., Chen W., Fan W. (2020). Protecting earthen sites by soil hydrophobicity under freeze-thaw and dry-wet cycles. Constr. Build. Mater..

[30] Ding L.-Q., Vanapalli S.K., Zou W.-L. (2021). Freeze-thaw and wetting-drying effects on the hydromechanical behavior of a stabilized expansive soil. Constr. Build. Mater..

[31] Salih W.T., Yu W., Dong X., Hao W. (2020). Study on stress-strain-resistivity and microscopic mechanism of red mud waste modified by desulphurization gypsum-fly ash under drying-wetting cycles. Constr. Build. Mater..

[32] Wang C., Guo C., Du X., Shi M., Liu Q., Xia Y. (2021). Reinforcement of Silty Soil with Permeable Polyurethane by Penetration Injection. Constr. Build. Mater..

[33] Yan Y., Wen B., Huang Z. (2017). Effect of soluble salts on shear strength of unsaturated remoulded loess in Lanzhou city. Rock Soil Mech..

[34] Ding J., Hong Z., Liu S. (2011). Microstructure study of flow-solidified soil of dredged clays by mercury intrusion porosimetry. Rock Soil Mech..

[35] Chen W.W., Zhang Q.Y., Liu H.W., Guo Z.Q. (2019). Feasibility of protecting earthen sites by infiltration of modified polyvinyl alcohol. Constr. Build. Mater..

[36] Du J., Zhou A., Shen S.L., Bu Y. (2022). Fractal-based model for maximum penetration distance of grout slurry flowing through soils with different dry densities. Comput. Geotech..

[37] Cheng J. (2019). The Research on Alkali-Activated Composite Grouting Material for Reinforcing Loess Foundation. Master’s Thesis.

[38] Meng L., Han L., Zhu H., Dong W., Li W. (2022). Study of the Effects of Compaction and Split Grouting on the Structural Strengthening Characteristics of Weakly Cemented Argillaceous Rock Masses. KSCE J. Civ. Eng..

[39] Xiang J., Liu L., Cui X., Yan H., Shi C. (2018). Effect of limestone on rheological, shrinkage and mechanical properties of alkali—Activated slag/fly ash grouting materials. Constr. Build. Mater..

[40] Li Z., Xiao J., Xie Q. (2021). Reliability analysis of the residual moment capacity of high-strength concrete beams after elevated temperatures. Struct. Concr..

[41] Wang T. (2017). The Theoretical Analysis and Experimental Study of Fracturing Grouting of Collapsible Loess. Ph.D. Thesis.

[42] Xing G., Liu C., Xuan W. (2020). Prediction of Unconfined Compression Strength for Saline-Alkaline Soils Mixed with Cement and Wheat Straw. Adv. Mater. Sci. Eng..

[43] Li J.D., Wang X., Zhang Y.J., Jiang D.J., Liu D.R., Wang J.L. (2021). Study on strength characteristics and mechanism of loess stabilized by F1 ionic soil stabilizer. Arab. J. Geosci..

[44] Gu K., Chen B. (2020). Loess stabilization using cement, waste phosphogypsum, fly ash and quicklime for self-compacting rammed earth construction. Constr. Build. Mater..

[45] Yang T., Zhang J., Zhang X., Zhang Q., Yin Z. (2020). Layered Grouting Technology Based on a Comprehensive Water-to-Cement Ratio for the Overlying Loess Stratum of Urban Shallow Tunnels. Adv. Civ. Eng..

[46] Yang R., Li K., Wang L., Bornert M., Zhang Z., Hu T. (2016). A micro-experimental insight into the mechanical behavior of sticky rice slurry-lime mortar subject to wetting-drying cycles. J. Mater. Sci..

[47] Vocka R., Galle C., Dubois M. (2000). Mercury intrusion porosimetry and hierarchical structure of cement pastes: Theory and experiment. Cem. Concr. Res..

[48] Zhu Y., Yu X., Lei Gao Chen J., Cotugno M.D. (2019). Unconfined Compressive Strength of Aqueous Polymer-Modified Saline Soil. Int. J. Polym. Sci..

